# Molecular Signaling in Pulmonary Vein Stenosis: Toward Lesion-Specific Phenotyping and Individualized Therapy

**DOI:** 10.3390/children13101304

**Published:** 2026-09-25

**Authors:** Mary Elizabeth Moss, Timothy Klouda

**Affiliations:** Division of Pulmonary Medicine, Boston Children’s Hospital, Harvard Medical School, Boston, MA 02115, USA; elizabeth.moss@childrens.harvard.edu

**Keywords:** pulmonary vein stenosis, neointima, pediatric, endothelial to mesenchymal transition, mechanotransduction

## Abstract

**Highlights:**

**What are the main findings?**

- Pulmonary vein stenosis (PVS) is a rare, progressive, fibrointimal proliferative disease of the pulmonary veins carrying a high morbidity and mortality despite antiproliferative strategies and aggressive catheter and surgical interventions.
- Emerging clinical and preclinical evidence implicates mechanosensitive signaling, endothelial-to-mesenchymal transition, and genetic factors in the development and progression of PVS.

**What is the implication of the main finding?**

- Mechanosensitive signaling and endothelial-to-mesenchymal transition pathways could prove to be novel therapeutic targets in pediatric PVS.
- Ongoing work to develop non-invasive methods of disease surveillance and to identify patient-specific biomarkers promises to improve the care of children with PVS.

**Abstract:**

Pulmonary vein stenosis (PVS) is a rare but life-threatening disease affecting young children, characterized by progressive narrowing of the pulmonary veins. Multimodal treatment, including surgical and catheter-based interventions combined with medical therapy, can improve survival; however, disease recurrence and progression are common. Currently, there is no therapy able to reverse existing disease, leaving lung transplantation the only option for patients with advanced, treatment-resistant disease. Studies using animal models and human tissue have identified several mechanisms that contribute to PVS, including mechanosensitive signaling and endothelial-to-mesenchymal transition. In parallel, advances in cardiac imaging and hemodynamic profiling of individual pulmonary veins have improved risk stratification and provided opportunities to modify treatment strategies. Despite these advances, the development of disease-modifying therapies able to improve patient outcomes will require a more thorough understanding of the underlying pathophysiology. In this review, we examine the histological features of PVS and summarize the cellular contributions and signaling pathways implicated in disease development. We will also explore the potential of integrating patient- and lesion-specific phenotypes and molecular signatures to guide individual treatment strategies, as well as the current limitations and future directions of PVS research.

## 1. Introduction

Pulmonary vein stenosis (PVS) is a progressive, life-threatening disease characterized by narrowing of one or more of the pulmonary veins, typically involving the extrapulmonary segment and venoatrial connection. Luminal narrowing in PVS causes obstruction and eventual occlusion of blood flow, leading to elevated pulmonary venous pressure, postcapillary pulmonary hypertension (PH), and, in severe cases, right-sided heart failure. The estimated incidence of PVS ranges from 0.0017% to 0.03% of the general pediatric population and is more common among infants and children with congenital heart disease (CHD) and/or chronic lung disease such as bronchopulmonary dysplasia (BPD) [1]. The true incidence of PVS is likely underestimated and may be increasing with improved survival and resuscitation of premature infants, advances in diagnostic testing, and a greater awareness of the disease among health care providers. Multimodal management, consisting of surgical and catheter-based interventions combined with medical therapy, remains the cornerstone of treatment; however, recurrence and progression are common. Even with current therapies, PVS is associated with significant morbidity and mortality, with the reported mortality ranging from 36% to 55% across different endpoints [2,3,4]. 

The clinical course of PVS is highly variable, and several features of PVS are associated with poor outcomes and the development of PH or right ventricular (RV) dysfunction [2,5,6,7]. The left-sided pulmonary veins are most likely to be affected, although the underlying mechanisms for this are unclear [3,8]. Patients with three or more stenotic vessels are more likely to experience adverse outcomes [6,7]. A younger age at the time of diagnosis also correlates with more aggressive disease, although any age-dependent molecular mechanisms have not been identified [7]. Lastly, emerging evidence suggests the presence of decompressing collateral veins correlates with decreased all-cause and PVS-related mortality, particularly in patients with bilateral disease [9].

Advances in cardiac imaging and the hemodynamic assessment of individual veins during cardiac catheterization have improved disease characterization, risk stratification, and longitudinal monitoring [10,11]. These tools can help identify patients at risk for poor outcomes and those who may benefit from more frequent surveillance and aggressive treatment. However, clinical phenotyping primarily stratifies a patient’s risk and characterizes the clinical manifestations of PVS without elucidating the underlying mechanisms driving disease progression. Furthermore, PVS is a heterogeneous disease, both among patients and across different vessels, and individual vein outcomes do not reliably correlate with patient outcomes. This suggests that vein- and patient-specific findings may reflect distinct biological processes that influence disease progression. 

At the tissue level, inspection of explant and autopsy specimens from patients with PVS demonstrates neointimal proliferation and the accumulation of myofibroblasts within the pulmonary veins. These changes can evolve over the course of the disease, contributing to luminal narrowing, stenosis, and eventual atresia of the affected vein. Despite these observations, the underlying mechanisms and signaling pathways driving these changes remain incompletely understood. Several mechanisms have been proposed in the pathogenesis of PVS, including the response of endothelial and mesenchymal cells to altered mechanical forces, endothelial-to-mesenchymal transition (EndoMT), and dysregulated extracellular matrix (ECM) remodeling. Defining how these contribute to PVS, the stimuli that promote their activation, and how their activity varies among patients and individual veins is critical. Before disease-modifying therapies can be proposed, a more comprehensive understanding of the cellular and molecular mechanisms leading to disease development is required. 

By integrating evidence from animal models that replicate PVS, histological studies, and risk stratification based on imaging and hemodynamic phenotyping, this review aims to establish a framework for patient- and lesion-specific approaches in PVS management. We will summarize the existing literature describing the molecular mechanisms and signaling pathways contributing to the development and progression of PVS, examine how molecular and lesion-specific phenotypes can inform treatment strategies, and outline current limitations and future directions for the field.

## 2. Clinical and Preclinical Features of PVS 

### 2.1. Classification

PVS can be classified as primary or secondary. Primary PVS develops in normally connected native pulmonary veins in the absence of prior surgical manipulation and is frequently associated with prematurity and chronic lung disease. Secondary PVS develops following surgical intervention involving the pulmonary veins, such as in repair of total anomalous pulmonary venous connection (TAPVC), hypoplastic left heart lesions, or Scimitar syndrome [12]. The disease can be further divided as either intraluminal, defined by the presence of neointimal proliferation within the vein, or obstructive, which is caused by extrinsic or anatomical abnormalities resulting in compression [12,13]. Rarely, PVS in pediatric patients can occur as an isolated or idiopathic condition in a full-term infant without pulmonary comorbidities and a structurally normal heart. Regardless of the underlying etiology, the histological findings of PVS are broadly similar, suggesting a shared signaling pathway that may be activated by different stimuli.

### 2.2. Histopathology

The histological appearance of stenosed pulmonary veins provides valuable insight into the molecular mechanisms underlying PVS. A study of 213 surgical specimens from 97 patients characterized the neointimal lesions as paucicellular, consisting of fibroblast-like cells within a myxoid matrix, collagen, and a lack of endothelial cells (ECs) [14]. Histological findings were similar between acquired and congenital PVS, suggesting a shared signaling program promoting disease progression. The paucicellular nature suggests the extensive neointimal thickening and intraluminal occlusion may not simply reflect EC proliferation but rather remodeling of the ECM. This is supported by lesional cells being largely negative for Ki-67 staining, arguing against uniform proliferation throughout the vessel wall [14,15]. 

The loss of an identifiable endothelial lining within the lumen of affected pulmonary veins, both proximal and extending distally into the lung parenchyma, suggests that ECs may lose their identity and acquire mesenchymal cell-like characteristics through EndoMT, contributing to the neointima and disease progression. Consistent with a myofibroblast-dominant phenotype, PVS specimens obtained after surgery exhibit strong smooth muscle actin (SMA) and muscle-specific actin staining [15]. Immunostaining of lesions has identified fibroblast growth factor receptor (FGFR), platelet-derived growth factor receptors alpha and beta (PDGFR-α, PDGFR-β), vascular endothelial growth factor (VEGF), and VEGF receptor 2 (VEGFR-2) in diseased veins, suggesting growth factor-driven signaling may be a key mechanism driving neointimal proliferation [15]. However, the presence of these molecules in end-stage tissue does not establish whether they drive disease activity or are rather a secondary response to disease progression. A summary of the clinical and basic science studies focusing on PVS can be seen in Table 1.

Beyond the pulmonary veins, lung tissue from patients with PVS also demonstrates pulmonary vascular changes consistent with those seen in PH. This includes increased smooth muscle cell (SMC) coverage of the interlobular septal veins and characteristic arterial hypertensive changes including medial hypertrophy and muscularization of pulmonary arterioles [16]. These distal changes likely contribute to increased pulmonary vascular resistance and PH observed in patients with advanced disease, suggesting venous obstruction in PVS extends beyond the location of the primary disease.

### 2.3. Preclinical Animal Models

Animal models have provided insight into PVS by reproducing the key hemodynamic and histopathological features observed in human disease [27]. The piglet pulmonary vein banding model replicates the hemodynamics and venous remodeling observed in patients, including SMC migration and progressive intimal fibrosis [28]. Another neonatal pig model of secondary PVS, achieved by cutting and resuturing the left lower pulmonary vein, likewise recapitulates SMA-positive neointimal proliferation [29]. The neonatal rat vein banding model also demonstrates neointimal remodeling while allowing for mechanistic investigation into the signaling pathways driving lesion formation [30,31]. Together, these models provide different strengths when studying PVS, with large animal models better representing clinically relevant hemodynamics and smaller animals allowing the integration of mechanistic and molecular studies. The potential to combine animal models of PVS with advanced transcriptomic studies such as single-cell RNA sequencing (scRNA-seq) and spatial transcriptomics provides investigators with a powerful platform to identify disease-associated cell populations and define the signaling pathways driving neointimal formation at single-cell and spatial resolution. A recent study performed proteomics of the pulmonary veins in the piglet pulmonary vein banding model of chronic pulmonary venous hypertension, identifying transforming growth factor beta (TGF-β) and mammalian target of rapamycin (mTOR) signaling as key pathways [32]. The use of animal models is particularly valuable for studying PVS, a disease where early-stage human tissue and patient-derived samples are limited and often difficult to obtain. At the same time, differences in vascular anatomy, lung development, and immune response between human and animal models must be carefully considered when interpreting findings and translating from bench to bedside. A summary of the preclinical animal models of PVS can be seen in Table 2.

Taken together, histopathological findings from both human and animal models converge on a shared downstream phenotype of myofibroblast-driven neointimal proliferation; however, the upstream mechanisms driving these changes may differ according to the type of disease and clinical context.

## 3. Molecular Signaling 

### 3.1. Mechanosensitive Signaling

In PVS, the pulmonary veins are exposed to altered mechanical forces secondary to obstruction and abnormal blood flow, which in turn leads to shear stress and increased venous pressures [36]. Increased blood flow from left-to-right shunting in patients with CHD is a known risk factor for PVS [3,5,8,37,38,39]. A computational fluid dynamics framework has been used to model time-averaged wall shear stress in the pulmonary veins across scenarios of varying disease severity [40]. The mechanism for the relationship between mechanical stress and vascular remodeling has not yet been fully elucidated in PVS. While mechanical alterations leading to transcriptional changes promoting abnormal cell proliferation, EndoMT, and vascular remodeling are suspected, direct evidence is limited, with insights coming largely from other vascular processes, including neointimal formation of the aorta and vascular graft failure [41,42]. 

The Hippo signaling pathway, largely through its downstream effectors Yes-associated protein (YAP) and transcriptional co-activator with PDZ-binding motif (TAZ), is a master mechanotransduction regulator that responds to shear stress and matrix stiffness [43]. In pulmonary arterial hypertension (PAH), YAP/TAZ activation in ECs and SMCs promotes abnormal proliferation, EndoMT, and an inflammatory response [44]. YAP/TAZ signaling also responds dynamically, as oscillatory flow activates the pathway and unidirectional laminar flow suppresses activity in human ECs [45,46]. Thus, the turbulent blood flow and shear stress in PVS may promote Hippo/YAP/TAZ activation, EC proliferation, and neointimal remodeling. This is further supported by in vivo data from the neonatal piglet pulmonary vein banding model of PVS, where YAP expression was upregulated in the neointima of stenosed pulmonary veins [35].

Piezo-type mechanosensitive ion channel component 1 (PIEZO1) in ECs and SMCs activates YAP/TAZ-regulated SMC proliferation, migration, and phenotypic shift, leading to neointima formation in human and murine models [47,48]. Vascular SMC-specific Piezo1 knockout has already demonstrated reduced neointimal hyperplasia in a guidewire-injury mouse model [47]. Additionally, mechanical stretch upregulates Piezo1 expression in fibroblasts [49]. The first study to characterize changes in PIEZO1 in relation to PVS performed bulk RNA sequencing on tissue from six pediatric patients with PVS [50]. This study identified PIEZO1 as a significantly upregulated gene in tissue from patients with aggressive PVS compared with non-aggressive PVS, in addition to differences in ECM regulation. While this highlights the potential for PIEZO1-related therapies to prevent progression, it is unable to determine PIEZO1 expression at a cellular level. Further studies leveraging scRNA-seq platforms will yield cell-specific insight into the role of PIEZO1 among various cell types. Due to the limited availability of patient-derived samples, future studies integrating transcriptional studies with rodent models of PVS are likely needed to shed additional light on the specific cellular and mechanistic roles of Piezo1 in PVS. 

Endothelial surface glycocalyx (ESG), composed of proteoglycans, glycosaminoglycans, and glycoproteins on the luminal surface of ECs, is a key mechanosensing component that translates hemodynamic forces into downstream signaling pathways, which in turn regulate vascular tone and inflammation [51]. Animal models have linked ESG components with endothelial nitric oxide production, in turn regulating vasodilation and downstream inflammatory pathways [52,53,54]. In a 3-D EC asymmetric stenosis culture model, shear stress led to matrix metalloproteinase-mediated ESG degradation and promoted inflammatory cell recruitment [55]. Wall shear stress has also been shown to change the composition of ESG, with laminar flow promoting hyaluronan production through glucose metabolism [56]. As the ESG represents the connection between fluid dynamic forces and EC signaling, changes in ESG composition and function in response to turbulent flow may represent an initiating event in the mechanosensitive cascade promoting neointimal formation.

### 3.2. Endothelial to Mesenchymal Transition

Unlike pulmonary arteries, which have a thick layer of smooth muscle, the media of pulmonary veins are relatively thin and sparsely populated with SMCs [57]. By contrast, the neointima in PVS stains robustly for the mesenchymal markers SMA and muscle-specific actin and also expresses the receptor tyrosine kinases PDGFR and FGFR [14,15]. Myofibroblast proliferation has been implicated as a contributor to vein restenosis in primary PVS [58]. EndoMT is a suspected source of myofibroblasts and driver of fibrointimal thickening. A mechanistic study using the neonatal piglet model of PVS demonstrated that after pulmonary vein banding, luminal ECs exhibit reduced platelet endothelial cell adhesion molecule-1 (PECAM1/CD31), von Willebrand Factor (vWF), and vascular endothelial cadherin expression, and the vessel wall upregulated levels of TGF-β, Smad4, and SMA [33]. Mechanical stress of the pulmonary veins is suspected to drive EndoMT in PVS, as oscillatory flow has been shown to potentiate latent TGF-β1 activation, providing a theoretical link between altered shear stress and EndoMT in PVS [59].

No lineage tracing or omics studies focusing on PVS have yet been published to confirm or characterize EndoMT. However, data from similar stenotic vessel diseases hint at potential mechanisms driving this process. Veins grafted into arterial flow environments, such as in coronary artery bypass grafts, undergo extensive remodeling, including neointimal proliferation, similar to the histopathology seen PVS [42]. EndoMT is integral to this process, with murine lineage tracing experiments revealing proliferation of endothelial-derived cells in affected venous grafts, which upregulate TGF-β and Smad/Slug signaling pathways and express SMA at high levels while downregulating CD31 [60]. However, these cells did not express the terminal SMC marker smooth muscle myosin heavy chain, supporting an intermediate phenotype rather than ECs obtaining a true SMC identity. The extent of EC contribution remains unclear. Lineage tracing experiments in vein graft models have attributed a substantial proportion of neointimal cells to EC origin [60]. Another fate-mapping study concluded that mature arterial and donor venous SMCs are the dominant source of myofibroblasts, with EndoMT-derived cells accounting for approximately 17% of the neointima and only in grafts with mild disease [61]. EndoMT is therefore regarded as one contributor among several to intimal proliferation, and its role in preclinical animal models of PVS is yet to be defined. Smad signaling and downstream mediators can be activated by regulators other than TGF-β [26]. In hepatic stellate cells, which are myofibroblast-like, PDGFR activates Smad2/3, promoting downstream gene expression [62]. Activin-A has also been shown to promote Smad2/3 phosphorylation and endothelin-1 expression in pulmonary artery ECs, which is thought to be a key contributor to PAH [63]. 

### 3.3. Mammalian Target of Rapamycin

A confluence of clinical and preclinical observations led to the identification of mTOR as a mechanistic target in PVS. The first evidence came from histopathologic staining of human PVS tissue, which was positive for PDGFR and VEGFR, suggesting a growth-factor-driven phenotype that may be amenable to antiproliferative therapy [14,15]. PDGFR, VEGFR, and other receptor tyrosine kinase signaling pathways converge on the phosphoinositide 3-kinase (PI3K)-AKT-mTOR pathway for downstream effector functions, thus providing an indirect mechanistic link for targeting mTOR signaling [64,65]. This connection was further demonstrated using the neonatal pig PVS model, which showed Ki-67-positive proliferation and mTOR phosphorylation in SMC-like cells of the venous neointima [29]. In this model, high-dose rapamycin-eluting films applied to the sutured pulmonary veins inhibited mTOR activation and delayed anastomotic stenosis, although the effect was transient and, at low doses, had no benefit. Similarly, in the neonatal rat pulmonary vein banding model, systemic sirolimus administration significantly reduced SMA-positive neointimal proliferation and RV wall thickness [31].

Pathways associated with EndoMT are also linked to mTOR through downstream TGF-β signaling. TGF-β2 drives EndoMT via both canonical Smad signaling and a non-canonical MEK/ERK, PI3K, and p38 (mitogen-activated protein kinase) MAPK pathways, each of which is required for Snail induction and EndoMT [66]. In non-canonical signaling, TGF-β transactivates PDGFR-β, thus facilitating downstream activation of PI3K, protein kinase B (Pkb/Akt), and mTOR [67]. Conversely, mTOR inhibition suppresses mesenchymal transition in models of pulmonary fibrosis, consistent with a feed-forward relationship between mTOR activation and mesenchymal identity [68]. The relationship between TGF-β, EndoMT, and mTOR signaling has been demonstrated in rat PAH models, where mTOR inhibition with rapamycin partially reversed the protein expression patterns of EndoMT and ameliorated PH [69].

### 3.4. Abnormal Angiogenesis

Histopathology of PVS lesions demonstrates widespread staining for VEGFR-2 and VEGF ligand [15]. This supports a potential role for neointimal angiogenic pathways in the formation of intimal lesions. This has been leveraged in clinical studies utilizing the VEGF inhibitor bevacizumab in patients with PVS [22,23]. The plexiform lesions of PAH are characterized by obliterative angiogenesis, a process thought to be influenced by VEGF signaling along with TGF-β and EndoMT pathways [70,71]. On the other hand, EC apoptosis and vascular pruning are associated with late-stage PAH [72]. mTOR can promote EC proliferation and angiogenesis [73], and mTOR family members are also upregulated in human PAH lesions [74]. In a similar fashion, dysregulation of angiogenic pathways may be a mechanistic factor in the progression of PVS and warrants further clinical and preclinical investigations.

### 3.5. Theoretical Links from Clinical Genetics: Cholesterol Synthesis and Chromosome 21

Genetic studies suggest additional mechanistic avenues that may predispose patients to PVS. Smith-Lemli-Opitz Syndrome (SLOS) is an autosomal recessive syndrome caused by mutations in 7-dehydrocholesterol- delta 7-reductase, the enzyme responsible for catalyzing the last step in cholesterol synthesis [75]. Aggressive forms of PVS have been described in infants with SLOS, raising the possibility that cholesterol synthesis defects may affect the pulmonary vasculature [76]. Infants with Trisomy 21/Down Syndrome (DS) are also at a higher risk for PH, a subset of which is attributable to PVS [77,78]. It has been postulated that the predisposition to PH, and likely PVS, is in part due to abnormal development of the pulmonary vasculature [79]. A recent study utilizing scRNA-seq of developing human DS lungs compared to healthy controls found widespread induction of ECM molecules in mesenchymal cells and activation of interferon signaling in ECs [80]. Because the interferon receptor gene cluster lies on chromosome 21, these findings raise the possibility of a regulatory program on that chromosome influencing pulmonary vascular development and predisposing patients with DS to PH and PVS.

## 4. Available Targeted Therapies 

### 4.1. Imatinib 

The first antiproliferative agent used as therapy for PVS was vinblastine combined with methotrexate. This combination was chosen based on the histopathological similarities between PVS and desmoid tumors [21]. However, in a clinical trial adverse effects led to treatment delays and interruptions, and efficacy was unable to be established. Following this study, based on the finding that cells from the neointima from patients with PVS express receptor tyrosine kinases, a single-arm pilot study was conducted to investigate the effects of PDGFR blockade in PVS with imatinib mesylate [22]. Imatinib, a tyrosine kinase inhibitor that acts through PDGFR-α/β, cellular Abelson murine leukemia viral oncogene homolog 1 (c-Abl), and proto-oncogene c-Kit (c-Kit), was proposed to inhibit the proliferative signaling driving myofibroblast-like lesions in patients with aggressive, multi-vessel disease [15,22]. In this study, imatinib was used as a monotherapy in patients with PVS associated with CHD, and VEGFR blockade with bevacizumab was added as an adjunct therapy for patients with primary PVS (i.e., not related to surgical correction of CHD). Patients in the study had an overall survival of 77% at 72 weeks, compared to a 1-year survival of 38% among patients with PVS and CHD in the vinblastine/methotrexate trial [21]. Furthermore, imatinib with or without bevacizumab was associated with fewer adverse effects compared to vinblastine and methotrexate in a similar cohort [21]. A sub-analysis of individual veins from the same study found that veins of patients who received a higher percentage of eligible drug doses required fewer reinterventions [23]. Together, these studies provided the initial evidence supporting inhibition of receptor tyrosine kinases as a mechanism-based therapeutic approach to PVS.

### 4.2. Sirolimus

Sirolimus is a macrolide with immunosuppressive, antiproliferative, and antiangiogenic effects [81]. It inhibits mTOR, a regulator of cell growth and proliferation, which in turn decreases T-cell activation and inhibits proliferation through arrest of the cell cycle [82]. While commonly used as maintenance immunotherapy in solid organ transplant recipients, its antiproliferative effects on myofibroblast neointimal proliferation and inhibition of vascular SMC migration and proliferation lead to it being used as a therapy to treat PVS [4]. The first study demonstrating the efficacy of sirolimus in patients with PVS came from Patel et al. in 2021 [4]. This study identified 67 patients who received treatment for moderate or severe primary or secondary PVS. Fifteen patients received sirolimus, while the remaining 52 did not and served as the control group. Survival was 100% in the sirolimus group compared with 45% in the control group. A separate retrospective study of 45 patients treated with sirolimus for ≥1 month found that the intervention rate fell from 5 to 1.7 interventions per year on therapy. Notably, this study had no external control group, as it compared intervention rates within patients before and during therapy. Estimated survival in this cohort was 77.9% five years after the diagnosis of PVS, and all six deaths occurred after sirolimus had been stopped, each in the setting of a preceding infection. No deaths occurred among the 25 patients who remained on therapy without interruption [18]. Overall, sirolimus is generally well tolerated, and adverse events requiring permanent discontinuation are rare [4,83]. Examples of reported side effects include serum lipid derangements, including hyperlipidemia and hypertriglyceridemia. Infections, including those from opportunistic pathogens, and delayed wound healing are potential complications of therapy due to the immunosuppressive actions of sirolimus.

Both systemic and locally delivered sirolimus have been shown to maintain venous patency after stent placement. A retrospective review of systemic sirolimus to prevent in-stent stenosis found that 85% of stents treated at the time of implantation were free of significant in-stent stenosis at a median of 102 days. Among stents treated after documented in-stent stenosis, the growth rate of restenosis slowed following initiation of sirolimus compared with pre-treatment [19]. Drug-eluting stents, with sirolimus or other agents that may inhibit neointimal proliferation, including paclitaxel, have overall shown promising results. The proposed mechanism of these stents include mTOR inhibition and, in the case of paclitaxel, cell cycle arrest, ultimately with the goal of arresting cellular proliferation within the vessel [4]. A study of 30 patients undergoing first catheter intervention found improved one-year vein patency with drug-eluting stents, but no association with patient survival. In the same cohort, reintervention was associated with improved patient survival [20]. Paclitaxel-coated balloons are increasingly used in PVS to potentially reduce neointimal proliferation after dilation; however, outcomes are yet to be formally investigated [37].

### 4.3. Combination Therapy: Imatinib and Sirolimus

Imatinib and sirolimus have each been associated with improved outcomes in patients with PVS. Combination therapy with both, in theory, could target complementary signaling pathways, leading to improved outcomes. A case series of three patients with aggressive PVS described patients treated with both drugs [83]. The authors report all three patients clinically stabilized after treatment and noted a decreased frequency of interventions. Vessel patency stabilized, with minimal to no intraluminal narrowing observed in the majority of the affected veins. Although including only three patients, this report supports more dedicated studies investigating the combination of imatinib and sirolimus to treat aggressive PVS.

### 4.4. Angiotensin Receptor Blockade

Losartan and valsartan are angiotensin receptor blockers used to manage hypertension and reduce stroke risk in select patients. In addition to inhibiting vasoconstriction and aldosterone-secreting effects, losartan can indirectly reduce TGF-β signaling, lowering circulating TGF-β1 in patients and in rodent models of renal fibrosis [24,84,85]. Accordingly, losartan has been shown to reduce EndoMT in other non-PVS cardiac models, including the myocardium of spontaneously hypertensive rats and human mitral valve ECs [86,87]. These findings provide a rationale that angiotensin II receptor blockade may have a role in PVS treatment. In the neonatal PVS pig model, losartan was shown to reduce intimal hyperplasia and improve the pulmonary artery-to-systemic blood pressure ratio [88]. Further mechanistic studies in the modified piglet banding model showed that losartan attenuated upstream intimal hyperplasia and reduced neointimal YAP expression, implicating involvement of the Hippo pathway [35]. A trial of losartan for the treatment of PVS (NCT02769130) was suspended following a national losartan recall. Separately, in a single-center study of 104 patients, valsartan started early after TAPVC repair did not reduce the incidence of postoperative PVS or reoperation but did significantly lower the risk of PVS progression [34]. These findings suggest the duration and timing of angiotensin receptor blockade may influence therapeutic outcomes. Further studies including patients with primary PVS are needed to clarify the role for angiotensin receptor blockade as a therapeutic agent.

## 5. Individual Molecular Profiling 

### 5.1. Circulating Biomarkers

Given the significant procedural burden of PVS for diagnostic and therapeutic purposes, noninvasive biomarkers to stratify disease severity would be of substantial clinical benefit. As of now, there is no serum biomarker to monitor PVS progression or response to treatment. However, data and studies focusing on PH suggest several potential candidates worth investigating. In one meta-analysis, high serum levels of brain natriuretic peptide (BNP) were associated with increased risk of death in pediatric patients with PAH [25]. While it may be used to trend RV dysfunction and PH associated with PVS, the use of serum BNP as a marker of early disease is likely limited. Additional studies have correlated PAH-associated mortality with serum troponin T and uric acid levels [89,90]. Serum galectin-3, a β-galactoside-binding lectin involved in inflammation and fibrosis, is elevated in PAH and connective tissue disease-associated PH, suggesting that this biomarker may be relevant to other pulmonary vascular diseases, including PVS [91]. The pathobiology of neointimal formation via EndoMT suggests that serum TGF-β or its downstream transcriptional targets may emerge as future biomarkers. Likewise, circulating matrix metalloproteinases could be indirect markers of vascular remodeling and ECM turnover. However, serum levels of these factors are not routinely measured in clinical practice, and their relationship to PVS disease activity is unknown. Further studies focused on the early molecular mechanisms of PVS are needed before identifying biomarkers that can predict severity, progression, and/or response to therapy.

### 5.2. Hemodynamic Measurements

In PVS, the severity of individual veins can vary within the same patient, and lesion-specific hemodynamic assessment is essential to accurately describe disease burden and prognosis. Cardiac catheterization can provide direct measurements of pulmonary vein obstruction and simultaneously enable the anatomical and physiological characterization of affected veins. In a prospective trial by Callahan et al., measurements during cardiac catheterization were obtained in 200 veins from 50 patients to create a multimodal risk stratification score [17]. The authors identified upstream luminal narrowing, ostial diameter, and upstream wall thickness measured with intravascular ultrasound as independent factors predicting worse 12-month outcomes. Using these measurements, a three-point invasive pulmonary vein score was developed to predict 12-month vein outcome, with transplant-free survival of the cohort being 92% at 12 months [17]. Furthermore, a higher postintervention gradient and wall shear stress were associated with worse individual vein outcomes. These findings highlight the importance of mechanical forces, not only as a marker of disease severity but also as biological drivers of lesion progression. In addition to intravenous ultrasound, optical coherence tomography (OCT) at the time of cardiac catheterization is emerging as a useful tool in evaluating individual vein parameters. OCT uses infrared light to produce high-resolution intravascular images. This technique was validated in a cohort of 11 pediatric patients with 34 pulmonary veins imaged; OCT was further utilized in a study of PVS patients treated with sirolimus, where it provided detailed visualization of in-stent regrowth [92,93]. Combining such measurements with markers of molecular pathways such as EndoMT, PIEZO1, or YAP/TAZ, either through serum biomarkers or cells collected during the procedure, could provide the framework for linking the hemodynamic environment of individual veins with their underlying phenotype and clinical trajectory. However, because cardiac catheterization is not without risk, complementary noninvasive approaches are needed to characterize the pulmonary vein anatomy and hemodynamics over time. 

### 5.3. Advanced Imaging Techniques

Multiple imaging modalities have been used in the diagnosis and monitoring of PVS, including echocardiograms, perfusion scans, and cross-sectional imaging. Chest computed tomography (CT) angiography with pulmonary vein enhanced protocols is well-suited to provide detailed information on pulmonary venous anatomy and assist in planning interventions. 3-D chest CT reconstruction can also identify ostial stenosis and upstream narrowing, features that correlate with worse outcomes [17,94,95,96]. The future use of advanced cross-sectional imaging and other modalities to determine disease severity may help mitigate the risks associated with cardiac catheterization and anesthesia in the future. For example, CT images have been combined with computational fluid dynamics to predict blood flow, calculating the time-averaged wall shear stress within the pulmonary vasculature and ventricles [36,40]. These approaches may be able to identify localized, abnormal mechanical environments promoting disease development and progression that are not apparent with other diagnostic testing, providing insight into the relationship between shear stress and disease development/progression.

In addition to chest CT angiography, 4-D MRI has been used to quantify blood flow through the pulmonary veins in PVS using a single acquisition, potentially limiting exposure to anesthesia and radiation while calculating velocity and hemodynamic parameters that echocardiography is unable to estimate [97]. Although experience with 4-D MRI in pediatric patients remains limited, this technique may provide a noninvasive approach with minimal exposure to radiation for serial evaluations of lesion-specific flow and hemodynamic assessment. 

Advanced echocardiographic techniques are also commonly used for non-invasive disease monitoring. While the pressure gradient of a pulmonary vein obtained via Doppler echocardiography does not correlate with measurements obtained during cardiac catheterization, flow peak velocity is a sensitive and specific measurement of pulmonary vein narrowing, and the presence of phasic flow is associated with a non-stenosed pulmonary vein [98]. Additional echocardiographic measurements including vein aliasing (wherein blood velocity is higher than the machine sampling rate) and pulmonary vein variability index are used in non-invasive assessments of disease, although the sensitivity and specificity of these measures are modest [99].

The combination of advanced imaging techniques with hemodynamic assessments obtained during cardiac catheterization can provide detailed information on individual pulmonary veins, further defining patients’ risk and disease severity. Together, these tools offer a non-invasive approach to hemodynamic phenotyping, which, when integrated with molecular profiling, can further guide individualized treatment decisions.

### 5.4. Genetics 

While no pathological variant has been proven to be causative of PVS, several syndromes and chromosome abnormalities are associated with an increased prevalence of PVS. In one cohort, 71 patients with PVS who had genetic testing performed revealed that 13 were diagnosed with DS, five with SLOS, and five with other genetic disorders: DiGeorge syndrome (22q11.2 deletion), Cat-Eye syndrome (partial tetrasomy or trisomy of chromosome 22q11.1–q11.2), mosaic Turner syndrome (45,X/46,XX), and a NOTCH1 pathogenic variant [100]. Twenty-four patients had variants of unknown significance identified on genetic testing, and an additional 24 had no gene variant identified, although it should be noted that genetic testing was limited to chromosomal microarray or specific gene panels. Investigations into other possible genetic underpinnings of PVS have begun to yield insights. 39% of identified patients with PVS in one study were born of twin pregnancies where the twin sibling was unaffected; however, this group included a mix of dichorionic and monochorionic twins, complicating analysis of the contribution of genetic factors [5]. One genome-wide study of a consanguineous Turkish family with four affected siblings mapped the first primary PVS locus to chromosome 2q35-2q36.1, although sequencing of 12 genes within the homozygous region identified no causative variant [101]. Together, these observations suggest a genetic susceptibility may be contributing to PVS in a subset of patients, although the mechanisms connecting these genetic conditions are unknown. As whole-genome sequencing becomes more accessible, broader genomic studies combining transcriptomic, histological, and lesion-specific phenotyping may reveal a causal genetic variant predisposing patients to PVS.

## 6. Future Direction and Limitations

A significant limitation in studying PVS is the lack of patient-derived samples across different stages of disease. While lung tissue from patients with PVS undergoing surgical resection or transplantation provides researchers with improved access to specimens for advanced transcriptomic and histological studies, these samples largely represent established and end-stage disease and are thus unable to provide insight into the early cellular and molecular changes that initiate and sustain PVS. Securing patient-derived samples early in the disease course, either during cardiac catheterizations or other less invasive approaches, is required to better understand disease development and progression. Due to the rarity of PVS, multicenter collaborations and the establishment of a coordinated biorepository and data-sharing infrastructure are needed to achieve such goals. The longitudinal collection of patients’ clinical, hemodynamic, and imaging data from individual veins will be particularly useful to overcome the rarity of PVS and investigate how molecular signaling changes over time in relation to disease severity.

While animal models replicate key features of PVS, including pulmonary hemodynamics and neointimal formation, they do not fully recapitulate the spontaneous and progressive nature of the disease, along with the environmental, genetic, and developmental factors at play. Additionally, they require a high degree of surgical skill and expertise. Regardless, preclinical models of PVS allow for advanced transcriptomic techniques, such as scRNA-seq and spatial transcriptomics, to study the early cellular contribution and signaling cascades promoting PVS. The development of a murine model of PVS that is able to replicate key features of human disease, while posing a technical challenge due to the small size of mice, could allow for the integration of cell-specific gene knockout and lineage tracing experiments, similar to those used in the investigation of PH [102,103,104]. The integration of animal models with molecular and clinical phenotyping of patients and individual veins will be necessary to identify, validate, and test potential disease-modifying therapies.

## 7. Conclusions

PVS is a highly lethal disease with a complex underlying pathogenesis. A growing body of evidence has identified altered mechanical forces, EndoMT, remodeling of the ECM, and dysregulated growth factor and vascular signals in the development and progression of PVS (Table 1), and emerging preclinical models promise further mechanistic insights (Table 2). The rarity of the disease itself, in addition to the scarcity of early-stage patient-derived samples, provides unique challenges in studying its underlying mechanisms. Advances in cardiac imaging and hemodynamic risk stratification during cardiac catheterization allow for phenotyping of individual veins and patients; however, these approaches cannot identify the underlying process driving aggressive and treatment-resistant lesions, emphasizing the need for ongoing molecular investigations into the pathobiology of PVS. Integrating clinical data with circulating biomarkers, early transcriptional changes, and potential genetic predispositions, along with utilizing preclinical animal models of PVS to identify and test mechanism-based therapies, will lead to a more complete understanding of PVS and how mechanical forces, cellular phenotypes, and signaling pathways interact over the course of the disease. These findings will be critical to moving PVS management from reactive to preventive and establishing new, disease-modifying therapies able to improve patient outcomes and survival.

## Figures and Tables

**Table 1:** Human studies in PVS and links to molecular mechanisms.

Description of Methods	Intervention	Sample Size	Key Findings	Molecular Mechanism	Ref
Retrospective analysis of surgically resected and autopsy PVS specimens	NA	213 specimens from 97 patients	Paucicellular myxoid lesions and loss of luminal endothelial lining	Fibroblastic proliferation and vascular remodeling	[14]
Case series of explanted PVS specimens	NA	7 cases	PVS lesions positive for SMA, PDGFR, and VEGFR staining	Myofibroblast and receptor tyrosine kinase signaling	[15]
Retrospective review of biopsy/explant/autopsy specimens	NA	33 patients	Thickening of interlobular septal vein and hypertensive changes in arteries	Vascular remodeling	[16]
Prospective single-center trial applying a standardized multimodal catheterization assessment	NA	200 veins in 50 patients	≥35% wall thickness measured with intravascular ultrasound is associated with worse vein outcome	Neointimal proliferation	[17]
Single-center retrospective study comparing sirolimus treatment vs. historical standard of care	Systemic sirolimus	15 treated, 52 controls	Sirolimus therapy improved survival (100% vs. 45%)	mTOR	[4]
Single-center retrospective study investigating sirolimus-treated patients compared to historical data	Systemic sirolimus	45 treated, no controls	77.9% 5-year survival from diagnosis, reduced intervention frequency (5 to 1.7 per patient-year on therapy)	mTOR	[18]
Single-center review of patients with PVS and history of stent placement treated with and without sirolimus	Systemic sirolimus	40 stents in 20 patients, divided into primary vs. secondary (after documented in-stent stenosis) prevention	85% of primary prevention stents without significant in-stent stenosis; slower restenosis rates in secondary prevention stents treated with sirolimus	mTOR, stent restenosis	[19]
Retrospective study evaluating reintervention rates after using drug-eluting stent compared to no stent and bare-metal stents	Drug-eluting stent (paclitaxel, everolimus, or zotarolimus)	9 with no stent, 5 bare-metal stent, 16 drug-eluting stent	Higher 1-year vein survival with drug-eluting stents, no association with patient survival; Reintervention associated with improved survival	Stent restenosis	[20]
Prospective study of chemotherapy in patients with PVS	Vinblastine and methotrexate	28 treated, 23 evaluated for response, no controls	38% 1-year survival among patients with PVS and CHD. Toxicity resulted in treatment delays	Neointimal proliferation	[21]
Single-arm pilot study investigating adjunct tyrosine kinase blockade in patients with PVS	Imatinib ± bevacizumab	48 treated, no controls	77% 72-week survival	PDGFR, VEGFR	[22]
Dose dependent secondary analysis of imatinib ± bevacizumab treatment in patients with PVS ^1^	Imatinib ± bevacizumab	46 patients, 182 veins	Veins in patients with higher percentage of eligible treatment doses required fewer interventions	PDGFR, VEGFR	[23]
Case series describing outcomes of patients treated with Imatinib and sirolimus	imatinib and sirolimus	3 treated	Clinical stabilization and improved vein diameter	PDGFR, mTOR	[24]
Single-center retrospective study after TAPVC repair (valsartan vs. protocolized control)	Valsartan	25 valsartan, 79 control	Lower risk of PVS progression in valsartan group	Angiotensin receptor	[25]

^1^ Re-analysis of data from ref. [26]. Abbreviations: CHD = congenital heart disease; mTOR = mammalian target of rapamycin; PDGFR = platelet-derived growth factor receptor; PVS = pulmonary vein stenosis; SMA = smooth muscle actin; TAPVC = total anomalous pulmonary venous connection; VEGFR = vascular endothelial growth factor receptor; NA: not applicable.

**Table 2:** Preclinical studies in PVS.

Description of Methods	Intervention	Sample Size	Key Findings	Molecular Mechanism	Ref
Neonatal piglet PV banding model with hemodynamic, morphometric, and biochemical analysis at 1, 3, and 6 weeks.	Piglet PV banding ^1^	20 banded, 18 sham, 12 non-operated	Increased PV compliance with elastin breakdown precedes SMC migration to the subendothelium and progressive intimal fibrosis. PA hypertension precedes rise in PV pressure	Elastin/collagen remodeling, SMC migration, recapitulation of human disease	[28]
Neonatal piglet bilateral PV banding ± stent implantation with hemodynamic and immunohistochemistry analysis at 7 weeks	Piglet PV banding ^1^	6 banded, 6 sham, 6 stented	Upstream intimal thickening with EndoMT features. Stent decompression partially reversed EndoMT but rapid in-stent restenosis followed	EndoMT, TGF-β	[33]
Neonatal Yorkshire piglet staged bilateral PV banding model treated with losartan	Piglet PV banding ^2^ and Losartan treatment	6 sham, 6 banded, 7 banded+losartan	Losartan reduced PA/systemic pressure ratio, decreased upstream intimal hyperplasia, and restored VE-cadherin expression	Angiotensin receptor, TGF-β	[34]
Infant pig PV banding model with repeat hemodynamics	Piglet PV cut-and-suture technique	1 week: 5 sham, 5 cut-and-suture; 4 week: 2 cut-and-suture; 8 week: 4 sham, 5 cut-and-suture	Increased Ki-67 and phosphorylated mTOR in SMC-like cells within the neointima. SMC dedifferentiation in upstream PVs.	mTOR, EndoMT	[29]
Infant pig PV banding model ± rapamycin-eluting film at anastomotic site	Piglet PV cut-and-suture PV obstruction model ± rapamycin-eluting film	15 pigs: 5 low-dose, 10 high-dose	High-dose rapamycin reduced p-mTOR-positive cells and delayed anastomotic occlusion	mTOR, EndoMT	[29]
Proteomics of male piglet PV banding model of chronic pulmonary venous hypertension validated against human autopsy tissue	Piglet PV banding ^3^	10 banded, 9 sham (proteomics performed on 6 banded and 6 sham)	PV remodeling driven by mTOR with EndoMT/TGF-β as top upstream regulator, mirrored in human specimens	EndoMT, mTOR, TGF-β	[32]
Effects of Losartan on PVS using the neonatal piglet vein banding model	Piglet PV banding + losartan	19 neonatal piglets, 7 sham, 6 banded, 6 banded+ losartan	Piglet vein banding model developed PH, intimal hyperplasia; Losartan treatment improved pathological changes and decreased YAP expression in intima	YAP activation, neointimal formation	[35]
Neonatal rat PV banding model with hemodynamic measurement by echocardiography/CT at postnatal day 21, histology at day 30	Neonatal rat PV banding ^4^	40 banded, 40 sham. 6 from each group underwent echocardiography and CT	Evidence of PH by echocardiography at postnatal day 21. Decrease in PV diameter by CT. Wall thickening and lumen narrowing in upstream PVs. Increase SMC layers in PVs.	Neointimal formation, recapitulation of human disease	[30]
Neonatal rat PV banding model	Neonatal rat PV banding ^1^	15 banded, 15 sham	Banded rats had increased proliferating SMA+ cells in neointima, increased ECM deposition, and decreased lumen diameter	Neointimal formation, EndoMT, SMC proliferation	[31]
Neonatal rat PV banding model ± systemic (intraperitoneal) sirolimus	Neonatal rat PV banding ^1^ ± sirolimus	60 banded + vehicle, 60 banded + sirolimus	Sirolimus improved survival, increased lumen diameter, reduced intimal thickness and SMA staining, and reduced RV free wall thickness	Neointimal formation, EndoMT, SMC proliferation	[32]

^1^ Bilateral pulmonary vein banding. ^2^ Right middle pulmonary vein left unbanded to serve as internal control. ^3^ Banding of the left anterior pulmonary vein and the posterior common pulmonary vein. ^4^ Only right-sided pulmonary veins banded. Abbreviations: CT = computed tomography; ECM = extracellular matrix; EndoMT = endothelial to mesenchymal transition; mTOR = mammalian target of rapamycin; PA = pulmonary artery; PH = pulmonary hypertension; PV = pulmonary vein; PVS = pulmonary vein stenosis; RV = right ventricle; SMA = smooth muscle actin; SMC = smooth muscle cell; TGF-β = transforming growth factor beta; VE-cadherin = vascular endothelial cadherin; YAP = Yes-associated protein.

## Data Availability

No new data were created or analyzed in this study. Data sharing is not applicable to this article.
